# A preliminary PET radiomics study of brain metastases using a fully automatic segmentation method

**DOI:** 10.1186/s12859-020-03647-7

**Published:** 2020-09-16

**Authors:** Alessandro Stefano, Albert Comelli, Valentina Bravatà, Stefano Barone, Igor Daskalovski, Gaetano Savoca, Maria Gabriella Sabini, Massimo Ippolito, Giorgio Russo

**Affiliations:** 1 Institute of Molecular Bioimaging and Physiology, National Research Council (IBFM-CNR), Cefalù, Italy; 2 Ri.MED Foundation, Palermo, Italy; 3 University of Palermo, Palermo, Italy; 4 Department of Physics and Astronomy, University of Catania, Catania, Italy; 5 Medical Physics Unit, Cannizzaro Hospital, Catania, Italy; 6 Nuclear Medicine Department, Cannizzaro Hospital, Catania, Italy

**Keywords:** Cancer, Active contour, Positron emission tomography, Biological target volume, Radiomics

## Abstract

**Background:**

Positron Emission Tomography (PET) is increasingly utilized in radiomics studies for treatment evaluation purposes. Nevertheless, lesion volume identification in PET images is a critical and still challenging step in the process of radiomics, due to the low spatial resolution and high noise level of PET images. Currently, the biological target volume (BTV) is manually contoured by nuclear physicians, with a time expensive and operator-dependent procedure.

This study aims to obtain BTVs from cerebral metastases in patients who underwent L-[^11^C]methionine (11C-MET) PET, using a fully automatic procedure and to use these BTVs to extract radiomics features to stratify between patients who respond to treatment or not. For these purposes, 31 brain metastases, for predictive evaluation, and 25 ones, for follow-up evaluation after treatment, were delineated using the proposed method. Successively, 11C-MET PET studies and related volumetric segmentations were used to extract 108 features to investigate the potential application of radiomics analysis in patients with brain metastases. A novel statistical system has been implemented for feature reduction and selection, while discriminant analysis was used as a method for feature classification.

**Results:**

For predictive evaluation, 3 features (asphericity, low-intensity run emphasis, and complexity) were able to discriminate between responder and non-responder patients, after feature reduction and selection. Best performance in patient discrimination was obtained using the combination of the three selected features (sensitivity 81.23%, specificity 73.97%, and accuracy 78.27%) compared to the use of all features. Secondly, for follow-up evaluation, 8 features (SUV_mean_, SUL_peak_, SUV_min_, SUL_peak_ prod-surface-area, SUV_mean_ prod-sphericity, surface mean SUV 3, SUL_peak_ prod-sphericity, and second angular moment) were selected with optimal performance in discriminant analysis classification (sensitivity 86.28%, specificity 87.75%, and accuracy 86.57%) outperforming the use of all features.

**Conclusions:**

The proposed system is able i) to extract 108 features for each automatically segmented lesion and ii) to select a sub-panel of 11C-MET PET features (3 and 8 in the case of predictive and follow-up evaluation), with valuable association with patient outcome. We believe that our model can be useful to improve treatment response and prognosis evaluation, potentially allowing the personalization of cancer treatment plans.

## Background

Radiomics has the potential to personalize patient management extracting relevant features from medical images for use in statistical models [1, 2]. Radiomics features can reflect the tumour pathophysiology and potentially, the evolution of the disease [3], for example through the evaluation of lesion heterogeneity [4, 5] known to cause treatment failure [6], improving the prediction of patient overall survival and/or outcome.

Radiomics aim is to construct a clinically relevant predictive or prognostic model, through the extracted features [7]. Precisely, when extracted from positron emission tomography (PET) images, these features, quantify biological characteristics with a potentially key role in the prediction of treatment response [8]. Moreover, physiological changes may occur before detectable anatomical changes, which makes the PET a valuable tool for an early treatment assessment. In addition, PET may be an excellent alternative to magnetic resonance imaging (MRI) or computed tomography (CT) in detecting unknown primary tumour thanks to high sensitivity for the detection of lesions [9]. Nevertheless, studies have demonstrated that there are numerous challenges in the extraction of quantitative parameters, such as the standardized uptake value (SUV) or the SUV normalized to lean body mass (SUL), from PET images [10]. In turn, the identification of the biological tumour volume (BTV), from which to extract appropriate and significant features, is of primary importance for the development of prognostic models. Radiomics only makes sense if the data extraction process is reproducible and repeatable. In addition, a precise tumour delineation, the process of defining the extent of the lesion in the image separating high uptake regions from background avoiding false-positives, is needed to avoid distortions in data extraction. This is a critical and still challenging issue due to the low spatial resolution and high noise level of PET images where boundaries between tissues are not always clearly defined [11]. Currently, the BTV is manually contoured by nuclear physicians. Although manual contouring seems like the most intuitive and easily implemented way of obtaining regions of interest (ROIs), it has many drawbacks. It is operator-dependent, time-consuming and labour-intensive. The high intra- and inter-operator variability associated with manual delineations gives less precise and mainly irreproducible results. For example, in the study proposed by Vorwerk et al. [12], the analysis of manual contouring data obtained by 18 physicians from 4 different departments highlighted a large inter-observer variability in tumour delineation, despite detailed instructions given to all for delineation process. A partial explanation for this high variability may be related to the partial volume effect [13]; lesion boundaries become blurred and unclear, making manual segmentation more challenging. This is an important limitation of several radiomics studies, i.e. [14], where ROIs were manually delineated. For this reason, many PET-based automatic segmentation methods have been proposed [15–18] but no consensus has been reached on the optimal delineation method recommending that no one single method can be used for general BTV delineation [19]. The large variability in the shape and texture of lesions makes difficult to generalize PET segmentation methods. In addition, in follow-up examinations (i.e. after radiotherapy or neoadjuvant chemotherapy), segmentation is challenging due to reduced metabolic uptake, lesion to background ratio and reduced tumour volume.

This study aimed to obtain BTVs from cerebral metastases in patients who underwent L-[^11^C]methionine (11C-MET) PET and to use these BTVs to extract radiomics features to stratify between patients who respond to radio-treatment [20] or not.

Methionine is a natural amino acid that shows a greater uptake by brain cancer cells, whereas it is low in normal cells. Precisely, 11C-MET uptake is mainly driven by the activation of the L-mediated and A-mediated amino acid transport across the blood-brain barrier. Although MRI remains the gold standard for diagnosis and follow-up after radiation therapy [21], 11C-MET PET can discriminate between cancer and healthy tissues, with great power of sensitivity and specificity. It has been reported that the extension of tumour cell invasion can be detected more clearly by 11C-MET PET rather than by CT or MRI approaches [22] providing complementary information to morphological imaging. As reported in [23], PET can be combined with MRI to provide specific information for defining the target volume for the radio-surgical treatment in patients with recurrent brain tumours, such as glioma, metastasis, and pituitary adenoma, to optimize target identification for infiltrating or ill-defined brain lesions. Other studies have shown that the 11C-MET PET specificity related to the extraction of the tumour volume is higher compared with MRI; for example, in the study reported by Grosu et al. [24], PET imaging was used for biological target delineation in 36 patients that showed a significantly longer median survival compared with the group of patients in which target volume was merely defined by MRI. Moreover, recent studies, conducted on primary brain tumours, propose an emerging PET approach to differentiate recurrent brain tumour from radiation necrosis using a radiomics-based model [25, 26].

For these reasons, in the management of brain disease, the integration of 11C-MET PET imaging in radiotherapy planning or follow-up evaluations represents a desirable step forward.

Starting from our previous study [27] where we proposed a semi-automatic method to segment lesions in whole-body [^18^F]fluoro-2-deoxy-d-glucose (18F-FDG) PET studies, we have implemented a fully automatic and operator-independent system for brain 11C-MET PET studies [28, 29]. The proposed system performs all segmentation steps automatically by individuating an optimal, operator-independent, initial ROI located around the metastasis on an automatically selected PET slice. So, once the ROI has been identified, it is fed to an enhanced local active contour (LAC) segmentation algorithm [27]. In the previous study [27], the proposed method outperformed another state of the art BTV segmentation method tested for comparison. In this study, the great sensitivity and specificity of 11C-MET in differentiating between healthy and malignant tissues is used to automatically identify an initial seed to start the BTV segmentation process, without any user intervention [28]. In other words, we show how this approach may be integrated into a fully automatic protocol for the segmentation of brain metastases in 11C-MET PET images. Based on these BTVs, 108 radiomics features were extracted using the open-source toolbox “Chang-Gung Image Texture Analysis” (CGITA) [30]. Since not all features carry important information, a novel statistical system based on correlation matrix and point-biserial correlation coefficient, was used to identify the most relevant features able to discriminate between responder and non-responder patients. Afterwards, Discriminant Analysis (DA) was used for patient classification.

In particular, 31 brain metastases for predictive evaluation, and 25 brain metastases for follow-up evaluation, have been considered to assess the suitability of the proposed system as a medical decision tool. PET studies and related structures containing BTV segmentations were imported in the CGITA toolbox to extract imaging features to investigate the predictive role of 11C-MET PET in the discrimination between patients who respond to treatment or not.

Summarizing, we aimed i) to propose a fully automatic segmentation system of BTVs with the consequent extraction of radiomics features, and ii) to implement a novel radiomics model to investigate the potential role of extracted features in the prediction of patient outcome in patients underwent 11C-MET PET examinations.

## Results

For basal evaluation, 15 men and 16 women with brain metastases (without other organ or/and bone metastases) were considered (men 62 ± 9y, women 53 ± 10y). Primary tumours were breast (22%), brain (32%) and lung cancer (46%). For follow-up evaluation, a subset of 10 men and 15 women with PET scan after gamma-knife or radio treatment was considered (men’s age 56 ± 8, woman’s age 53 ± 10). Gamma-knife was used to safeguard the surrounding healthy brain tissue in the case of brain disorders inaccessible for a conventional surgery allowing accurate external irradiation (with a single, high dose and steep dose gradient) and minimizing doses given to adjacent critical brain structures. In the other cases (i.e. for big tumours), stereotaxic radiotherapy using a conventional linac-based system was performed.

The median time between PET scans was 6 months. A clinical evaluation was carried out by our medical staff to differentiate between responder and non-responder patients using MRI studies after the treatment for the 6 patients without follow-up PET study, and MRI and PET studies after the treatment for the remaining 25 patients. In particular, patients with progression or stable disease were considered as non-responders, patients with partial or complete response were considered responders.

**Fig. 1:**
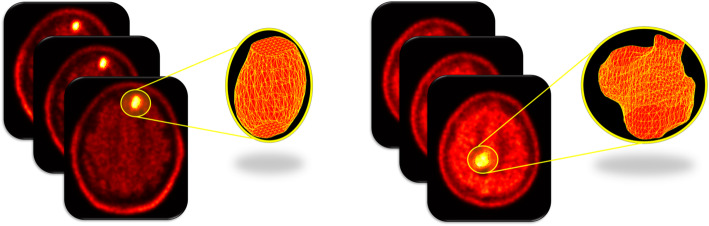
Two examples of three-dimensional volume reconstructions

**Fig. 2:**
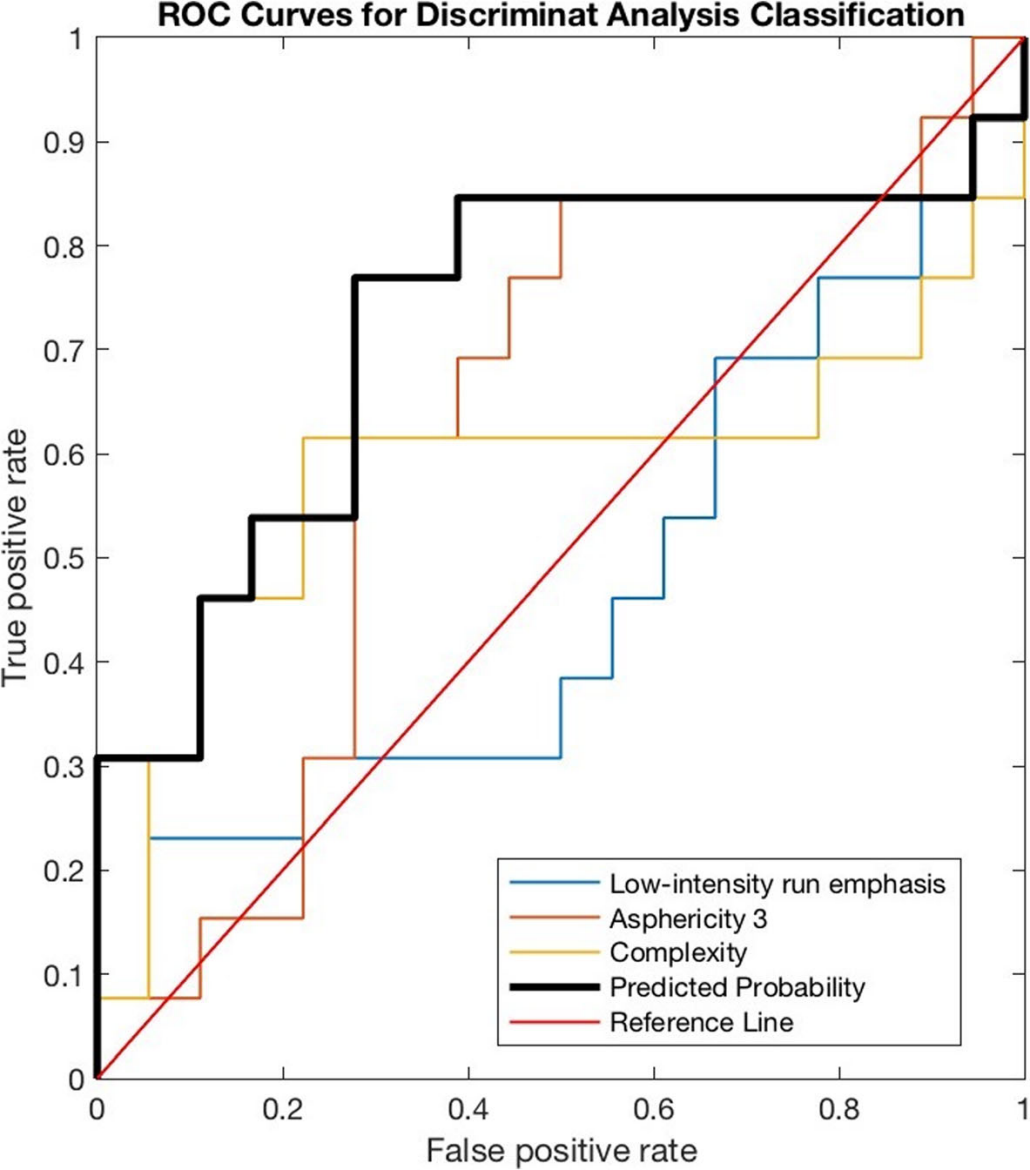
ROC curves for predictive evaluation. The black bold line represents the combined predicted probability by using the 3 selected features (AUC = 0.73; 95% C.I. 0.52–0.93)

CGITA toolbox [30], using 11C-MET PET images and automatic extracted BTVs, allowed the extraction of 108 radiomics features for each selected metastasis. BTV segmentation examples are shown in Fig. 1. For predictive evaluation, 3 features (asphericity, low-intensity run emphasis, and complexity) were able to discriminate between responder and non-responder patients after feature reduction and selection. Precisely, asphericity is a measure of the deviation from a spherical shape, while the low-intensity run emphasis is a textural feature based on a grey level run (a set of consecutive, collinear voxels having the same grey level value). The length of the run is the number of voxels in one direction and reflects the size of texture elements [31]. The complexity refers to the visual information content of a texture. So, a texture is considered complex if the information content is high: this occurs when there are many patches in the texture with different average intensities [32]. Best performance in DA classification was obtained using the combination of the 3 selected features with sensitivity 81.23%, specificity 73.97%, precision (or positive predictive value) 82.94%, negative predictive value 71.95%, error 21.73% and accuracy 78.27%, compared to the use of all features. All results are shown in Table 1. Corresponding receiver operating characteristic (ROC) curves are shown in Fig. 2.

**Table 1:** Comparison of performance in DA classification for predictive evaluation

	Sensitivity	Specificity	Precision	Negative Predictive Value	Error	Accuracy	
**Selected features**	81.23%	73.97%	82.94%	71.95%	21.73%	78.27%	
**All features**	69.90%	59.43%	69.67%	59.69%	34.63%	65.37%

**Fig. 3:**
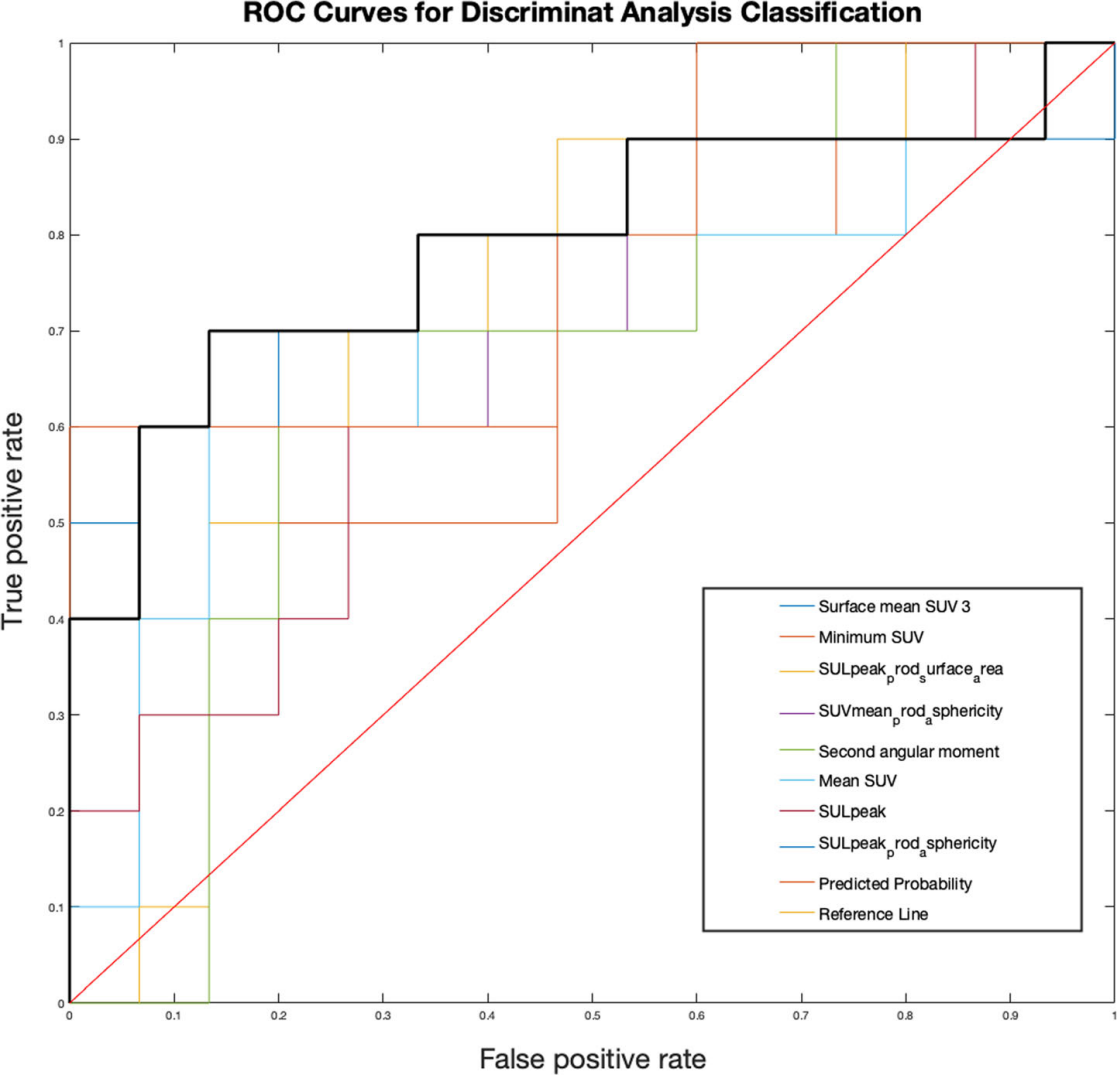
ROC curves for follow-up evaluation. The black bold line represents the combined predicted probability by using the 8 selected features (AUC = 0.79; 95% C.I. 0.59–1.00)

For follow-up evaluation, 8 features (SUV_mean_, SUL_peak_, SUV_min_, SUL_peak_ prod surface area, SUV_mean_ prod sphericity, surface mean SUV 3, SUL_peak_ prod sphericity, and second angular moment) were selected with optimal performance in DA classification (sensitivity 86.28%, specificity 87.75%, precision 92.10%, negative predictive value 80.22%, error 13.43%, and accuracy 86.57%) outperforming the use of all features in the DA classification. The various SUV and SUL related features are intensity-based metrics coupled with the surface area or the asphericity. The second angular moment is a measure of texture homogeneity or uniformity [33]. Corresponding results and ROC curves are shown in Fig. 3 and Table 2, respectively.

**Table 2:** Comparison of performance in DA classification for follow-up evaluation

	Sensitivity	Specificity	Precision	Negative Predictive Value	Error	Accuracy
**Selected features**	86.28%	87.75%	92.10%	80.22%	13.43%	86.57%
**All features**	94.98%	67.55%	75.49%	92.96%	19.16%	80.84%

## Discussion

To date, prognosis and treatment response evaluation in the oncological field is still challenging. There is a crucial need to identify biomarkers predictive of patient outcomes to improve personalized treatment. Radiomics has emerged as a potential solution to this issue, providing a multitude of features from biomedical images, i.e. PET images. One advantage of radiomics is that it uses diagnostic images that are available already without requiring additional exams. Despite some encouraging results, several challenges still need to be addressed. Reproducibility and robustness of radiomics studies involving PET images are influenced, among other things, by the choice of the delineation method used to identify the BTV [34]. The lesion segmentation process must be reliable and repeatable. This can only be achieved by using computer-assisted methods. For this reason, we propose a fully automatic segmentation system that eliminates any user intervention, thus increasing result repeatability [29]. The proposed system determines an initial ROI around the lesion, differently than in the original semi-automatic system [27]. Besides, we incorporate in the system the results of the DA classifications to discriminate between patients with brain metastases able to respond to treatment or not. Our ultimate goal is to aid in creating personalized therapy.

After automatic BTV delineation and radiomics feature extraction, the applied DA classification yields promising results. In particular, firstly we implemented a novel statistical system based on correlation matrix and point-biserial correlation coefficient to recursively eliminate features, to select the most relevant ones. Secondly, we used the DA classification to stratify patients. Best performance in classification was obtained using the combination of the selected features (3 and 8 features for predictive and follow-up evaluation, respectively) compared to the use of all 108 features, improving the specificity for more accurate risk stratification in the brain metastases.

Our results showed that 11C-MET PET radiomics has a great predictive value (sensitivity = 81.23%, specificity = 73.97%), comparable with other 11C-MET PET studies [35, 36]. In addition, 11C-MET PET showed great potential in follow-up evaluation after radiotherapy (sensitivity and specificity were 86.28 and 87.75%, respectively), thus potentially helping the clinicians to assess treatment outcomes. In the latter case, we would like to underline that the 8 selected features include PET parameters (i.e. SUV and SUL) that are usually taken into consideration in PET studies that do not perform radiomics analysis to assess treatment follow-up, i.e. [37–39]. However, our radiomics analysis did not use the 8 selected features individually as in the above-mentioned studies, but they were combined to classify patients (see Fig. 3).

**Fig. 4:**
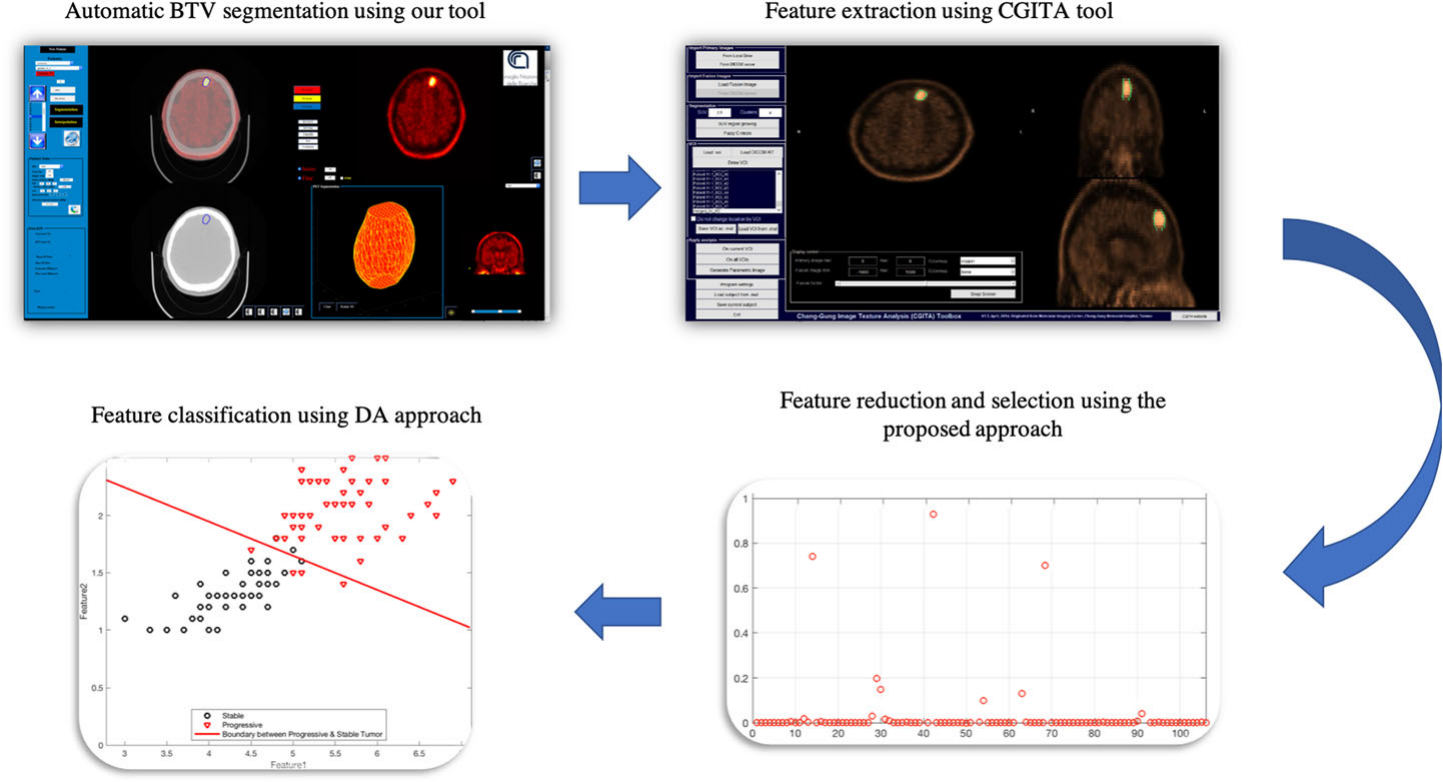
The proposed workflow from the fully automatic BTV segmentation process to DA classification to discriminate between patients who respond to treatment or not

A major limitation of the proposed study is the relatively small dataset used for the training and validation step. So, a future study with more data is expected to yield even better results improving the prediction of patient outcome. Finally, considering that the proposed system and the CGITA toolbox have been implemented in the Matlab environment running on a standard PC, the whole system (from the BTV segmentation to DA classification, see Fig. 4) could be easily integrated into the clinical setting as a built-in tool in PET workstations. This allows clinicians to use the BTV information both for radiotherapy treatment planning, prognosis and treatment response evaluation to improve personalized medicine.

## Conclusions

In our study, the proposed system was able i) to extract 108 features for each automatically segmented lesion and ii) to select a sub-panel of 11C-MET PET features (three in the case of basal evaluation and eight in the case of follow-up evaluation) with valuable association with patient outcome. We believe that our model can be useful to improve treatment response and prognosis evaluation, potentially allowing the personalization of cancer treatment plans.

## Methods

Patients with brain metastases underwent 11C-MET PET/CT scan and radiotherapy treatment, including the Leksell Gamma Knife (Elekta, Stockholm, Sweden), were considered in this retrospective analysis. Leksell Gamma Knife is a stereotactic radio surgical device able to treat brain metastases that are inaccessible for a conventional surgery allowing accurate irradiation to target through a metal helmet.

Functional and anatomical imaging modalities provide complementary data that can be integrated to provide a better diagnosis and to improve the effectiveness evaluation of oncological treatments. For this reason, the PET is always combined with CT or MRI to provide co-registered functional and anatomical images. Anatomical imaging also provides crucial data for attenuation correction of PET images. Metastasis segmentation in PET images was performed off-line without actually influencing the treatment protocol or patient management. No sensitive patient data were accessed. As such, after all patients were properly informed and released their written consent, the institutional hospital medical ethics review board approved the present study protocol.

### Image acquisition

Although the 18F-FDG is the most commonly used radiotracer in PET studies, its specificity and sensitivity are notably reduced in the brain region. As a matter of fact, 18F-FDG is a glucose metabolism tracer whose distribution involves cells according to the glucose transport mechanism and, consequently, it is not limited to malignant tissue, i.e. 18F-FDG PET can be limited by unspecific uptake in inflammatory benign lesions [40]. In [41], 18F-FDG shows limited clinical value when comparing brain tumour volume defined by metabolic imaging with the corresponding volume defined by PET/MRI images due to low contrast between tumour and healthy tissue in 18F-FDG PET images. Vice versa, 18F-FDG PET may be useful in distinguishing common enhancing malignant brain tumours, particularly high grade and low-grade gliomas and lymphoma [42–44]. Nevertheless, a high 18F-FDG uptake in surrounding healthy tissue limits its use for the imaging of a large fraction of primary and recurrent tumours [45]. For example, glioblastoma shows glucose uptake higher than in anaplastic astrocytoma that, vice versa, shows hypo-metabolism. This difference could be explained by the presence of necrosis [46]. In conclusion, 18F-FDG PET often poses a challenge in the identification of treatment-induced necrosis, edema, inflammation, and pseudo-progression. For this reason, other PET radiotracers have been developed, i.e. 11C-MET that shows excellent sensitivity and specificity in the detection of brain metastases [21, 22, 35]. Thanks to the injected 11C-MET radiotracer, metastasis appears as a hyperintense region. Unfortunately, 11C-MET PET is not widespread in clinical practice due to very short half-life of ^11^C (around 20 min) requiring a short interval between synthesis, injection, and acquisition. Consequently, only the medical centres which have an onsite cyclotron can utilize 11C-MET radiotracer. Alternatively, [^18^F]fluoro-ethyl-l-tyrosine (18F-FET) significantly correlates with brain tumour cell density and proliferation [40] and it can be distributed to PET centres without a cyclotron unit on site (^18^F half-life = 110 min).

In our study, 11C-MET PET/CT imaging scans were performed at Cannizzaro Hospital in Catania (Italy) in compliance with the standard brain oncological protocol in use in this institution. Patients fasted for at least 4 h before the examination performed on Discovery 690 scanner (General Electric Medical Systems, Milwaukee, WI, USA), and successively were intravenously injected with MET. The PET/CT oncological protocol started 10 min after the injection. The PET protocol included a SCOUT scan at 40 mA, a CT scan at 140 keV and 150 mA (10 s), and 3D PET scans (6 min per bed position). ‘Ordered Subset Expectation Maximization’ with two-iterative process was used as a 3D reconstruction algorithm. Images were reconstructed to a 256 × 256 matrix with a grid spacing of 1.17 mm^3^ and a thickness of 3.27 mm^3^. The CT scan performed contextually to the PET imaging was used for attenuation correction.

### The fully automatic segmentation method

In the proposed method, PET images were pre-processed as previously described by our group [47]. Precisely, the body-weight SUV, the most widely used PET parameter, was used to convert PET images into SUV unit (g/ml) images. Successively, to obtain a fully automatic BTV segmentation system starting from the one proposed [27], our algorithm performs all segmentation steps automatically by individuating an optimal, operator-independent, initial ROI located around the tumour on an automatically selected PET slice. By taking advantage of the great sensitivity and specificity of 11C-MET radio-tracers in discriminating between healthy and tumour tissues, the system identifies the PET slice containing the maximum SUV (SUV_max_) in the whole PET dataset avoiding any user intervention. Consequently, the SUV_max_ voxel is used as a target seed for a region growing segmentation [18] to automatically identify a ROI containing the lesion. It is worth noting that the region growing algorithm is used only to obtain a rough estimate of the lesion boundary. This initial operator-independent ROI is input to the next component of the system, an enhanced LAC segmentation algorithm [48], as extensively explained [27]. Despite the LAC method is able of locally widening or tightening around the lesion boundary, a stopping criterion has been implemented to prevent wrong segmentations when a free disease slice is reached. This because the LAC algorithm is driven by the image properties rather than by an inherent knowledge of whether the tumour is present. In other words, the LAC is automatically stopped when a free disease PET slice is reached [27]. In this way, the proposed system becomes fully automatic for the segmentation of brain metastases in 11C-MET PET images [29]. In the case of multiple brain metastases, each lesion is independently processed. A different local maximum (SUV_max-j_, with j = 1:n) is identified for each lesion. By design, the first BTV contains global SUV_max_. The delineation iterative procedure ends when the SUV_max_ of the currently processed lesion is less than 2 g/ml. However, the user will receive a warning message in case of multiple lesions and will be able to stop the process to avoid false-positive occurrences (healthy tissues with SUV_max_ > 2 g/ml).

### Radiomics features extraction

After automatic BTV delineations, 11C-MET PET studies and related structures containing volumetric segmentation were imported in the open-source CGITA toolbox [30] to extract radiomics features from each lesion and to investigate the potential application of radiomics analysis in patients with brain metastases. The extracted radiomics features were grouped into first-order, second-order, and higher-order features. First-order features derive from the histogram of PET voxel intensities such as SUV_max_ and SUV_mean_, SUV normalized to lean body mass (SUL), total lesion proliferation (TLP), median, skewness, kurtosis, variance, entropy, etc. Second-order textural features provide information about the regional spatial arrangement of the voxels such as their homogeneity, and contrast simulating the human perception of tumours in PET images. Higher-order features provide information on local collinear voxels with the same grey level. Specifically, grey levels inside each volume were re-sampled in 64 quantization levels and 9 texture matrices in 3D with 26-voxel connectivity. Texture features were computed on grey level co-occurrence matrix (7 indices), voxel alignment matrix (11 indices), neighbourhood grey level difference matrix (5 indices), grey level size zone matrix (11 indices), normalized grey level co-occurrence matrix (6 indices), texture spectrum matrix (2 index), texture feature coding matrix (4 indices), texture feature coding co-occurrence matrix (8 indices), and neighbourhood grey level dependence matrix (5 indices) [30, 49]. A total of 108 imaging features were calculated for each metastasis, considering additional 49 SUV indices (see Table 3). For follow-up evaluation, we considered the feature variations (Δ) in sequential PET scans normalized to baseline examinations:

1 $$ \Delta \left(\%\right)=100\ \mathrm{x}\ \left(\mathrm{post}\hbox{-} \mathrm{treatment}\ \mathrm{feature}\ \mathrm{value}-\mathrm{baseline}\ \mathrm{feature}\ \mathrm{value}\right)/\mathrm{baseline}\ \mathrm{feature}\ \mathrm{value} $$

**Table 3:** Radiomics features extracted from each brain metastasis

Parent matrix	Feature measure
**Cooccurrence matrix** (7 features)	Second angular moment, contrast, entropy, homogeneity, dissimilarity, Inverse difference moment, correlation
**Voxel-alignment matrix** (11 features)	Short-run emphasis, long-run emphasis, intensity variability, run-length variability, run percentage, low-intensity run emphasis, high-intensity run emphasis,
	low-intensity short-run emphasis, high-intensity short-run emphasis, low-intensity long-run emphasis, high-intensity long-run emphasis
**Neighborhood intensity difference matrix** (5 features)	Coarseness, contrast, busyness, complexity, strength
**Intensity size-zone matrix** (11 features)	Short-zone emphasis, large-zone emphasis, intensity variability, size-zone variability, zone percentage, low-intensity zone emphasis, high-intensity zone emphasis, low-intensity short-zone emphasis, high-intensity short-zone emphasis, low-intensity large-zone emphasis, high-intensity large-zone emphasis
**Normalized cooccurrence matrix** (6 features)	Second angular moment, contrast, entropy, homogeneity, inverse difference moment, dissimilarity
**Texture spectrum** (2 features)	Max spectrum, Black-white symmetry
**Texture feature coding** (4 features)	Coarseness, homogeneity, mean convergence, variance
**Texture feature coding cooccurrence matrix** (8 features)	Second angular moment, contrast, entropy, homogeneity, intensity, inverse difference moment, correlation, variance, code similarity
**Neighborhood gray-level dependence** (5 features)	Small-number emphasis, large-number emphasis, number nonuniformity, second moment, entropy
**SUV indices** (49 features)	Minimum SUV, SUVmax, mean SUV, SUV variance, SUV SD, SUV skewness, SUV kurtosis, SUV skewness (and with bias corrected), SUV kurtosis (and with bias corrected), TLG, tumor volume, entropy, SULpeak, Surface area, Asphericity 1and 2 and 3, Surface mean SUV 1 and 2 and 3 and 4, Surface total SUV 1 and 2 and 3 and 4, Surface SUV entropy 1 and 2 and 3 and 4, Surface SUV variance 1 and 2 and 3 and 4, Surface SUV SD 1 and 2 and 3 and 4, Surface SUV NSR 1 and 2 and 3 and 4, SUVmean prod asphericity, SUVmax prod asphericity, Entropy prod asphericity, SULpeak prod asphericity, SUVmean prod surface area, SUVmax prod surface area, Entropy prod surface area, SULpeak prod surface area

Due to the redundancy, heterogeneity and uncertainty of the information represented by radiomics features, the use of these data efficiently and reliably is challenging [50]. For this reason, we used the correlation matrix and point-biserial correlation coefficient for feature reduction and selection, while DA [51] was used as a machine learning method for feature classification.

Briefly, the correlation matrix was used to identify the most relevant features able to discriminate between responder and non-responder patients. Matrix columns correspond to extracted features, rows correspond to observations. The first step was to identify, for each feature, the highly correlated features (correlation coefficient greater than 0.9). For each highly correlated feature, the point-biserial correlation coefficient was calculated between the feature and gold standard (responder vs non-responder patient). The point-biserial correlation coefficient is used in the case of dichotomous variables (in our case, the gold standard). Then, we selected the feature with a higher point-biserial correlation coefficient among the highly correlated features. The remaining features were deleted. This step was repeated for each feature of our dataset. The second step was to recalculate the point-biserial correlation coefficient for each feature identified in the first step and the gold standard. The features with a higher coefficient were selected (point-biserial correlation coefficient > 0.25) and area under the curve (AUC) and confidence interval of selected features were calculated. The above-mentioned correlation coefficient thresholds (0.9 and 0.25, respectively) were empirically determined to provide the best performance on the present dataset with the classifier results.

After the feature selection process, DA was used to identify the linear combination of selected features that characterize or separate two or more classes of objects. We trained DA using a random partition method to split data into training and validating sets. The grouping was made so that both the training and validation sets maintained the same responder and non-responder patient status percentage of the original dataset. In particular, k-fold cross-validation was used to partition the whole dataset in k parts of equal numerosity to validate the classification method. For each fold, k-1 of the data was used as a training set and the remaining of the data as a validation set. In other words, the whole dataset is splitting into k equal subsets, and the holdout method is repeated k times. Each time, one of the k subsets was used as the validation set, and the other k-1 subsets were used as a training set. Training vectors were labelled as responder or non-responder patients. Then the average error across all experiments was computed. In this way, overfitting and asymmetric sampling were avoided increasing the precision of final results. In this study, k = 5 has been empirically determined through the trial-and-error method (k range: 5–15, step size of 5).

## Data Availability

The datasets used and/or analysed during the current study are available from the corresponding author on reasonable request.

## Abbreviations

AUC: Area under the curve
BTV: Biological target volume
CGITA: Chang-Gung image texture analysis
CT: Computed tomography
DA: Discriminant analysis
18F-FDG: [^18^F]fluoro-2-deoxy-d-glucose
18F-FET: [^18^F]fluoro-ethyl-l-tyrosine
LAC: local active contour
11C-MET: L-[^11^C]methionine
MRI: Magnetic resonance imaging
PET: Positron emission tomography
ROC: Receiver operating characteristic
ROI: regions of interest
SUL: Standardized uptake value normalized to lean body mass
SUV: Standardized uptake value
TLP: Total lesion proliferation
